# Complete Genome Sequence of an Atypical Porcine Pestivirus Strain, GX01-2018, from Guangxi Province, China

**DOI:** 10.1128/MRA.01440-18

**Published:** 2019-02-07

**Authors:** Yanwen Yin, Kaichuang Shi, Wenchao Sun, Shenglan Mo

**Affiliations:** a Guangxi Center for Animal Disease Control and Prevention, Nanning, China; b Institute of Virology, Wenzhou University, Wenzhou, China; Indiana University, Bloomington

## Abstract

We report the complete genome sequence of an atypical porcine pestivirus (APPV) strain named GX01-2018 that was isolated in Guangxi Province, China, from a suckling piglet showing congenital tremor. The whole genome consisted of 11,565 bp and shared 83.4% to 98.2% nucleotide identities and 91.9% to 99.1% amino acid identities with other APPV strains from different countries.

## ANNOUNCEMENT

Atypical porcine pestivirus (APPV), a member of the genus *Pestivirus* of the family *Flaviviridae*, was first isolated in the United States in 2015 (1) and then was proved to be the causative agent of type A-II congenital tremor (CT) (2 – 5). So far, APPV has been reported in the United States, the Netherlands, Germany, Austria, Spain, China, Hungary, Brazil, England, and Sweden (2 – 14), showing that APPV is widely distributed around the world. Infected piglets are characterized by generalized body shaking with variable degrees of hypomyelination in the brain and spinal cord. The prevalence of CT varies from 16% to 100% within affected litters. The case fatality rate reached 30% in Brazil (8), and the number of weaned piglets per sow decreased by more than 10% due to CT in Austria (5). So, further evaluation of the hazard of APPV is needed. Here, we report the complete genome sequence of an APPV strain named GX01-2018.

The GX01-2018 strain was isolated in April 2018 in Guangxi Province, China, from a suckling piglet showing CT. Homogenized tissue samples of brain, spleen, and lymph nodes were analyzed by reverse transcription (RT)-PCR to determine APPV presence. To determine the complete genome sequence, RT-PCR was used with 8 pairs of specific primers to amplify 8 overlapping fragments encompassing the open reading frame (ORF) of the GX01-2018 strain (15). The 5′ untranslated region (5′ UTR) and 3′ UTR were acquired by rapid amplification of cDNA ends (RACE). The PCR products were purified, cloned into a pMD18-T vector, and sequenced with an ABI 3730XL sequencer (TaKaRa, China). The sequences of the above-mentioned fragments were assembled into the full-length sequence with SeqMan software (DNAstar, Inc. USA).

The complete genome sequence of the GX01-2018 strain was 11,565 bp in length, with a 5′ UTR of 378 nucleotides (nt), followed by a single large ORF and a 3′ UTR of 279 nt. The ORF was 10,908 nt in length and encoded a polyprotein of 3,635 amino acids. The polyprotein was composed of 12 proteins, including N^pro^, C, E^rns^, E1, E2, P7, NS2, NS3, NS4A, NS4B, NS5A, and NS5B. Homology analysis revealed that the GX01-2018 strain shared 83.4% to 98.2% nucleotide identities and 91.9% to 99.1% amino acid identities with other APPV sequences available in GenBank. Phylogenetic analysis showed that the GX01-2018 strain, together with the strains JX-JM01-2018A01 from China (GenBank accession number MG792803), GZ01/2016 from China (KY475592), AUT-2016_C from Austria (KX778724), Bavaria_S5/9 from Germany (KU041639), and NL1 from the Netherlands (KX929062), was located in one branch, whereas the GX04/2017 strain (MH102210), which was also isolated from Guangxi province, together with the strains HBtl1701 from China (MF377344) and 000515 from the United States (KR011347), was located in another branch (15). The results suggested that APPV has a large amount of genetic variation in Guangxi Province, China. The genomic data of the GX01-2018 strain will provide a better understanding of the molecular epidemiology and genetic diversity of APPV.

### Data availability.

The complete genome sequence of the GX01-2018 strain is available in GenBank under the accession number MH715893.
